# Multi-scale mechanistic modelling of the host defence in invasive aspergillosis reveals leucocyte activation and iron acquisition as drivers of infection outcome

**DOI:** 10.1098/rsif.2021.0806

**Published:** 2022-04-13

**Authors:** Henrique AL Ribeiro, Luis Sordo Vieira, Yogesh Scindia, Bandita Adhikari, Matthew Wheeler, Adam Knapp, William Schroeder, Borna Mehrad, Reinhard Laubenbacher

**Affiliations:** ^1^ Division of Pulmonary, Critical Care, and Sleep Medicine, Department of Medicine, University of Florida, Gainesville, FL, USA; ^2^ Department of Psychiatry, University of Florida, Gainesville, FL, USA; ^3^ Department of Pathology, University of Florida, Gainesville, FL, USA; ^4^ Center for Quantitative Medicine, School of Medicine, University of Connecticut, Farmington, CT, USA; ^5^ Kitware Inc., Clifton Park, NY, USA

**Keywords:** *Aspergillus fumigatus*, innate immune response, iron homeostasis, computational model

## Abstract

Aspergillus species are ubiquitous environmental moulds, with spores inhaled daily by most humans. Immunocompromised hosts can develop an invasive infection resulting in high mortality. There is, therefore, a pressing need for host-centric therapeutics for this infection. To address it, we created a multi-scale computational model of the infection, focused on its interaction with the innate immune system and iron, a critical nutrient for the pathogen. The model, parameterized using published data, was found to recapitulate a wide range of biological features and was experimentally validated *in vivo*. Conidial swelling was identified as critical in fungal strains with high growth, whereas the siderophore secretion rate seems to be an essential prerequisite for the establishment of the infection in low-growth strains. In immunocompetent hosts, high growth, high swelling probability and impaired leucocyte activation lead to a high conidial germination rate. Similarly, in neutropenic hosts, high fungal growth was achieved through synergy between high growth rate, high swelling probability, slow leucocyte activation and high siderophore secretion. In summary, the model reveals a small set of parameters related to fungal growth, iron acquisition and leucocyte activation as critical determinants of the fate of the infection.

## 1. Introduction

Invasive aspergillosis is a human infection with increasing incidence, related to the use of immunosuppressive therapies, such as cancer chemotherapy and immunosuppression medications [1]. More recently, it has also been observed that 10% to 14% of critically ill patients with COVID-19 developed invasive aspergillosis [2,3]. Mortality remains high, 30–60% in recent surveys [4], despite advances in diagnostics and therapy. Increasing triazole resistance in this infection [5] has raised the spectre of a ‘perfect storm’ [6] in an increasing population of susceptible individuals with a diminished repertoire of treatment options.

The research presented here was motivated by the search for host-centric interventions in immuno-compromised patients that can be used in combination with antifungal treatments. An important mechanism in innate immunity is the sequestration of iron from pathogens, a nutrient critical for nearly all organisms. A well-established literature supports the concept that the ‘battle over iron’ is characteristic of the host’s attempt to attenuate microbial growth during many infections [7]. Iron is particularly relevant to the pathogenesis of aspergillosis [8]. The iron sequestration feature of the innate immune response involves several intertwined processes that unfold across spatial and temporal scales. This makes it challenging to assess the effect of perturbations of individual mechanisms on infection dynamics. A computational model that captures the key mechanisms, broadly reflects the underlying immune biology, and is well-validated can play an essential role in hypothesis generation and the discovery of emergent properties of the immune response.

Several models related to respiratory *Aspergillus* infections and their pathology have been previously published. For example, agent-based models have shown the necessity of chemotactic signals for proper fungal clearance [9,10]. Our own work includes a model of the innate immune response to *A. fumigatus*, showing that a key determinant of infection is the range at which macrophages can detect the fungus [11], and an intracellular regulatory network linking iron metabolism to oxidative stress in a fungal cell [12]. The model presented here is parameterized entirely with information from the literature, rather than through data fitting, and is validated by showing that it can recapitulate a wide range of experimental data and features reported in the literature that were not used in its construction, as well as experimental data generated for the purpose of model validation. The model was then used to identify major drivers of the growth of fungal burden, providing potential targets for intervention.

## 2. Methods

### 2.1. A computational model of the immune response to invasive aspergillosis

The model is an agent-based model of invasive pulmonary aspergillosis scaled to a mouse lung, the experimental system used in this study, focusing on the ‘battle over iron’ between host and fungus. We first describe its main components.

#### 2.1.1. Space and time

A three-dimensional space representing a small portion of a mouse lung is divided into a discrete grid of one thousand voxels (10 voxels in each of three dimensions), representing a total volume of 6.4 × 10^−2^ μl. Each voxel has an edge length of 40 μm (6.4 × 10^−5^ μl). Cells and molecules have no space coordinates other than the voxel in which they are located at a given time. This approach is similar to that used in the general immune modelling platform C-IMMSIM [13]. The space has periodic boundary conditions, and simulated time progresses in discrete steps of 2 min.

#### 2.1.2. Molecules

The model includes five kinds of molecules: cytokines (IL-6 (interleukin 6), IL-10 (interleukin 10), TGF (transforming growth factor *β*1), TNF-*α* (tumour necrosis factor *α*), CXCL2 (chemokine (C-X-C motif) ligand 2), CCL4 (chemokine (C-C motif) ligands 4)), a siderophore (triacetylfusarinine C (TAFC)), iron-carrier molecules (transferrin and lactoferrin), iron and the hormone hepcidin. The cytokines are subject to a half-life of 1 h [14–20]. Furthermore, all the molecules are subject to a constant exchange between the simulated volume and the serum (system). Iron in this model is used only as a temporary buffer for the transference of iron between dying cells (i.e. macrophages, *A. fumigatus*) and iron-carrier molecules.

The concentration of a molecule in one voxel is called the local concentration, and we will refer to the concentration across the whole simulated space as the global concentration. By contrast, the serum concentration (i.e. outside the simulator) is the systemic concentration. Equation (2.1) determines the flux of molecules between the serum and the simulated (local) space. For the cytokines lactoferrin and TAFC, we assume as a simplification that the systemic concentration *x*_system_ is zero. Therefore, these molecules are constantly flowing in the direction of the serum, increasing their decay rate. The systemic level of hepcidin and transferrin is dynamically calculated as follows:

2.1 $$y=(x-x_{\mathrm{system}})\times e^{-k_{\mathrm{turn}}\times t}-x_{\mathrm{system}},$$

where *x*_system_ is the molecule’s systemic concentration (see terminology above), *x* is the local concentration, *k*_turn_ is the turnover rate and *t* is the time-step length (2 min).

Equations (2.2) and (2.3) compute the systemic levels of hepcidin and transferrin. Note that the result of the first equation (2.2) feeds into the second one (2.3). However, the first equation needs the systemic levels of IL-6 as input. However, in this simulator, we only have the local and global levels of IL-6. According to Goncalves *et al.* [21], a reasonable estimate of the systemic level of IL-6 is one-half of its global level. The equations are

2.2 $$\begin{array}{rl} & {\mathrm{Log}}_{10}({\mathrm{Hepcidin}}_{\mathrm{systemic}})={\mathrm{hep}}_{\mathrm{int}}+{\mathrm{hep}}_{\mathrm{slope}}\times {\mathrm{Log}}_{10}\left(\frac{IL6_{\mathrm{global}}}{2}\right), \end{array}$$

and

2.3 $$\begin{array}{rl} & {\mathrm{Transferrin}}_{\mathrm{systemic}}=Tf_{\mathrm{int}}+Tf_{\mathrm{slope}}\times {\mathrm{Log}}_{10}({\mathrm{Hepcidin}}_{\mathrm{systemic}}). \end{array}$$

These equations are based on data from Tabbah *et al.* [22], correlating systemic levels of IL-6 to systemic levels of hepcidin, and Moran-Lev *et al.* [23] correlating transferrin and hepcidin. To ensure biologically meaningful values, we only evaluate these equations if IL-6_global_ > 1.37 × 10^−10^ M and ${\mathrm{Hepcidin}}_{\mathrm{systemic}}>10^{-8}\;M$.

All these molecules, except iron, diffuse through space, modelled using the alternating direction implicit method with periodic boundary conditions [24]. The rationale for periodic boundary conditions is that the simulation covers a small area amid a large infected area. Therefore, the concentration of molecules across all boundaries should be similar.

#### 2.1.3. Host cells

**Figure 1.**
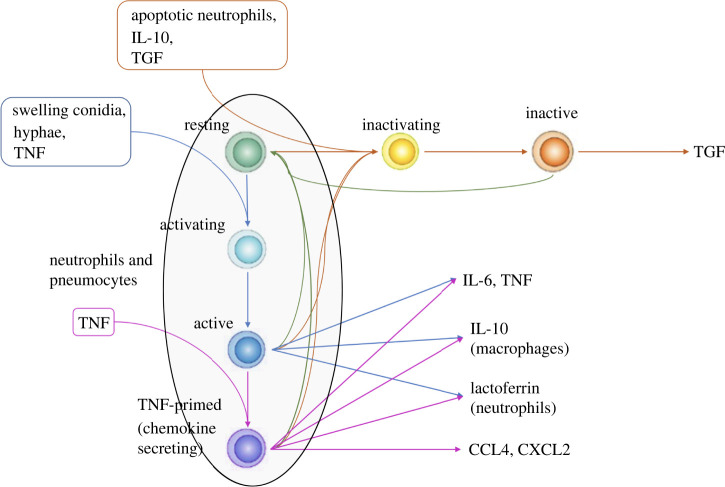
Figure showing host cell state changes. The paragon host cells in the model are the macrophages. Therefore, this figure represents the whole state space of a macrophage. The other cells (neutrophils and type II pneumocytes) have a subset of the states that macrophages have (see the area in the ellipse). By default, host cells are in a resting state. Swelling conidia, hyphae or TNF causes them to transition to an activating (intermediate) state and then to the active state. Active host cells secrete TNF, IL-6 and IL-10. Extra priming with TNF makes host cells secrete chemokines as well (CCL4 and CXCL2). A macrophage will return to the resting state after 6 h (180 iterations) in the absence of a continuous stimulus [25,26]. Apoptotic neutrophils, IL-10 or TGF-*β*1 cause macrophages (including activated macrophages) to transition to an anti-inflammatory TGF-*β*-secreting state. Active macrophages (blue and purple) can kill hyphae whereas resting and anti-inflammatory ones cannot.

There are three types of host cells: type II pneumocytes, macrophages and neutrophils. The host cells can assume several different states and transition from one to another upon interacting with other agents or molecules. Figure 1 shows the graph of host cell states. We introduce the states ‘activating’ and ‘inactivating’ to model the delay between signal and phenotype change. For a review of macrophage activation/inactivation, see Duque & Descoteaux [27] and Gordon [28].

For neutrophils, the state change as implemented here is not entirely realistic. For simplicity, the neutrophils in the model do change state, but this change has a minimal impact on their actions within the model (figure 1).

Leucocytes are free to move through the space and can be recruited as well. Recruitment is done according to equation (2.4):

2.4 $$n=\frac{k_{r}\times X}{k_{d}}\times \left(1-\frac{N}{K}\right),$$

where *N* is the current number of cells in the simulator, *K* is the carrying capacity, *k*_*r*_ is the global recruitment rate, *k*_*d*_ is the dissociation constant of the chemokine, *X* is the global amount of the chemokine (i.e. the average concentration of chemokine in the simulator) and *n* is the average number of cells to be recruited. This number is used by a Poisson random number generator to decide how many cells will be recruited. Macrophages and neutrophils have half-lives of 24 h and 6 h, respectively [29,30]. The quantity of cells in the simulator is a balance between the number of cells recruited according to equation (2.4) and the number of cells that die. Macrophages are recruited by CCL4 and neutrophils by CXCL2.

In the absence of chemokines, cells move randomly, while in their presence, they tend to move to the voxels with higher chemokine concentrations. The rate of movement is constant, and the cells will, on average, traverse a fixed number of voxels per time step. In the presence of chemokines, each voxel receives a weight according to equation (2.5):

2.5 $$w_{i}=1-e^{-(x_{i}/k_{d})},$$

where *x*_*i*_ is the chemokine concentration in neighbouring voxel *i*, *w*_*i*_ is the corresponding weight of this voxel and *k*_*d*_ is the chemokine dissociation constant. The cell will then move to a neighbouring voxel (*v*_*i*_) with probability proportional to the voxel weight (*p*_*i*_ ∝ *w*_*i*_).

#### 2.1.4. Aspergillus

In the model, *Aspergillus fumigatus* has three life stages: resting conidia, swelling conidia and hyphae. The hyphae are more or less continuous structures divided by septae [31]. Each of these subdivisions is a multinucleated cell-like structure, referred to as a hyphal cell for simplicity.

In previous work, a dynamic gene regulatory network of iron uptake by *Aspergillus fumigatus* was developed [12], in the form of a Boolean network, that is used here as a component model, with minor adjustments, as follows. While the original network had a TAFC node activating the node LIP, representing the labile iron pool, comprised of metabolically available iron, we modified it as follows. Instead, when TAFC is activated, the cell secretes TAFC. Later on, if the cell is expressing the siderophore receptors, the cell takes up TAFCBI (TAFC bound to iron) from the environment. The TAFCBI uptake increases the cell’s total iron pool. The LIP node then becomes a function of the iron pool. Using equation (2.6), we activate LIP if the iron pool is high. Resting conidia do not produce or take up TAFC in our model. We update the Boolean network every 30 min (every 15 iterations of the tissue-scale model) [32]. For simplicity, we consider only TAFC as extracellular siderophore. However, note that *A. fumigatus* also produces fusarinine C and recent evidence indicates that it may also secrete ferricrocin [33,34].

In simulations, *Aspergillus fumigatus* starts out as a pool of resting conidia; after 4 h these start swelling with a half-life of 6 h (see electronic supplementary material, table S1)—that is, half the conidia swell after 6 h. Beyond that, it takes 2 h until they become able to grow into hyphal cells. However, growth is controlled by LIP. That is, if LIP is off, hyphae cannot develop, consistent with the known importance of iron for hyphal growth. Hyphal swelling and germination is a complex phenomenon determined by a cascade of intracellular signalling (see Baltussen *et al.* [35] for a review). We represent this cascade in a simplified manner with first-order probability.

Although hyphal growth is a continuous process, the model uses a discrete approximation. A tip cell can produce another tip cell (elongation), while a sub-tip cell can form a 45° branch (subapical branch) [31,36] with a 25% probability. Other cells cannot originate new cells unless their neighbours are killed, and they become tip or sub-tip cells again.

#### 2.1.5. Interactions

Electronic supplementary material, table S2, displays the interactions between all cell types and molecular species. Note that cells/agents may interact with each other in more than one way, depending on their state. Due to software engineering considerations, we consider the secretion of molecules a kind of interaction between a cell and a molecule. Therefore, interactions between molecules and cells can also be of two kinds: receptor binding (interaction in the biological sense) and secretion. Note that, in electronic supplementary material, table S2, macrophages need to be active in order to kill hyphae, while neutrophils do not. For agents to interact with each other, they must be in physical proximity, i.e. in the same voxel.

Interactions between cells and molecules follow equation (2.6):

2.6 $$p=1-e^{-(x/k_{d})},$$

where *x* is the molecule concentration, *k*_*d*_ is its dissociation constant and *p* is the probability of receptor activation. Likewise, interactions between molecules (i.e. reactions) follow the Michaelian equation (equation 2.7):

2.7 $$v=\frac{K_{\mathrm{cat}}\times S1\times S2}{K_{M}+S1}.$$

In equation (2.7), *v* is the reaction velocity, *K*_*M*_ is the concentration of reactant *S*1 such that the velocity is one-half of the maximum velocity, known as Michaelian constant, and *K*_cat_ is the catalytic constant.

#### 2.1.6. Scaling from the simulated space to the whole mouse lung

To scale from the simulated space to the whole lung, we considered the volume of a pair of mice lungs and the fraction of the lungs that *Aspergillus* infects. We considered a volume of 1 ml for a pair of lungs [37] and that the infection occupies approximately one-third of this volume based on experiments of inhalation of particles [38]. Therefore, to scale from the small simulated space to the whole lung, we multiply by 5028. This number is an approximation and considers only the infected areas of the lung. Nevertheless, these are fair approximations for the numbers of leucocytes and *Aspergillus*.

However, the number of epithelial cells and the initial number of macrophages are evenly distributed across the lung and do not depend on the infection. Therefore, to calculate the number of these cells for initializing the model, we only considered the lung volume and the simulated area’s volume.

### 2.2. Experimental methods

#### 2.2.1. Neutrophil depletion and induction of aspergillosis

All experimental animal studies were performed in compliance with the National Research Council Guide for the Care and Use of Laboratory Animals, the United States Animal Welfare Act and the Public Health Service Policy on Humane Care and Use of Laboratory Animals. Protocols were approved by the Institutional Animal Care and Use Committees of the University of Florida.

Invasive aspergillosis was induced using previously published protocols [39,40]. *Aspergillus fumigatus* strain 13073 (ATCC, Manassas, VA, USA) was grown on Sabouraud dextrose agar plates for 10–14 days, and conidia were harvested in 0.1% Tween-80 in PBS and filtered through sterile gauze. Conidial concentration was then determined using a hemocytometer.

Wild-type C57BL/6 mice were purchased from The Jackson Laboratory (Bar Harbor, ME, USA) and maintained under pathogen-free conditions in the animal facilities of the University of Florida. Sex-matched male and female eight-week-old mice were used in the experiments. Neutrophils were transiently depleted with an intraperitoneal injection of 400 μg of anti-Ly6G antibody (clone 1A8, BioXcell) in 0.5 ml saline or an equivalent amount of isotype control antibody (rat IgG2a, Clone 2A3, BioXcell) 1 day before the *Aspergillus* challenge. The following day, animals were anaesthetized with a cocktail of ketamine and xylazine and intratracheally inoculated with 7 × 10^6^ *Aspergillus* conidia in 30 μl saline. At each designated timepoint, six animals per group were euthanized using heparinized anaesthetic overdose. The pulmonary vasculature was perfused with PBS containing 0.5 mM EDTA, bronchoalveolar lavage performed as previously described [41], or lungs were removed en bloc for flow cytometry.

#### 2.2.2. Flow cytometry

Mouse lung flow cytometry was performed as described in [41]. Briefly, lungs were digested in a mixture of 200 μg ml^−1^ DNaseI and 25 μg ml^−1^ Liberase TM (Sigma, St Louis, MO, USA) for 30 min at 37°C. The digested lungs were serially passed through 70 and 40 μm filters to collect the single-cell suspension. After red blood cell lysis, cells were counted, and 1.5 × 10^6^ cells were stained with a fixable APC Cy-7 conjugated live dead stain (Thermo Fisher, Waltham, MA) in PBS for 20 min. After washing with FACS buffer, cells were incubated with anti-CD16/32 (Fc block, clone 93; eBioscience, San Diego, CA) and stained with PerCP-conjugated anti-CD45 (30-F11), FITC-conjugated anti-CD11b (M1/70), PE-conjugated CD64 (X54-5/7.1), PECy7-conjugated anti-CD11c (N418), V450-conjugated anti-MHCII (I-A/I-E), APC-conjugated anti-CD24 (M1/69), BV605-conjugated anti-Ly6g (1A8), BV711-conjugated Ly6c (HK 1.4), and Texas Red-conjugated Siglec F (E50-2440) (all from Thermo Fisher). Flow cytometry data were acquired using 14 colour BD Fortessa (BD Biosciences, San Jose, CA). Some 500 000 events/samples were acquired and analysed with FlowJo software v. 9.0 (Tree Star Inc., Ashland, OR).

#### 2.2.3. Bronchoalveolar lavage fluid cytokine measurement

BAL (bronchoalveolar lavage) IL-6 and CXCL2 levels were measured using commercial ELISA kits (Invitrogen), as per manufacturers’ instructions.

## 3. Results

### 3.1. Model validation

**Figure 2.**
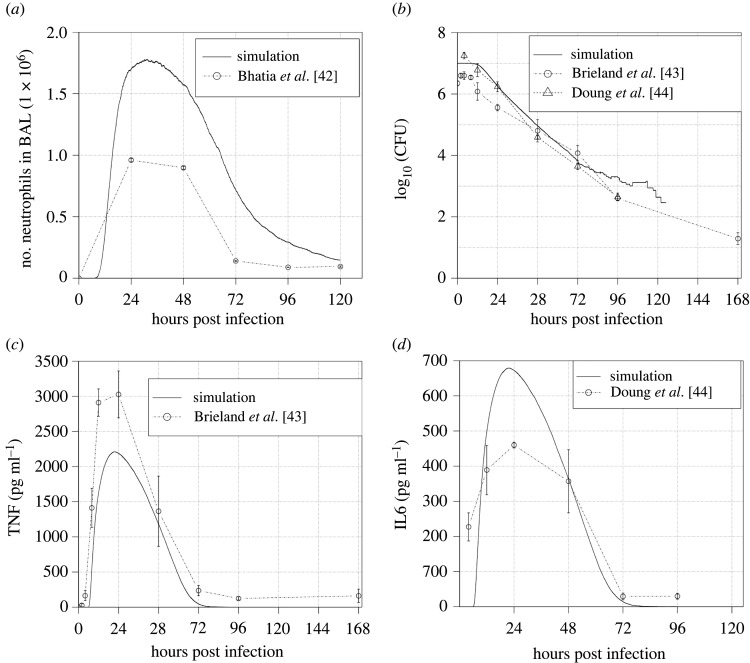
Comparison of simulated data with data reported in the literature. The solid lines are the average of the same cohort of 36 simulations with the same parameters and starting with an average of 1920 conidia, 15 macrophages and 640 epithelial cells. (*a*) Simulated time series of neutrophils and a time series reported by Bhatia *et al.* [42]. (*b*) Simulated time series of conidia and time series reported by Brieland *et al.* [43] and Doung *et al.* [44]. (*c*) Simulated time series of TNF and time series reported by Brieland *et al.* [43]. (*d*) Simulated time series of IL-6 and time series reported by Doung *et al.* [44].

This model was completely parameterized with data from the literature (see electronic supplementary material), thereby ensuring a much broader validity than could be obtained through parameter fitting to a small collection of experimental time-course measurements. We have validated the model in two ways. Firstly, we show that it provides a good qualitative fit with several time-course datasets in the literature that were not used in model calibration. We used a collection of papers that report time series of critical variables present in the model, such as neutrophils, TNF-*α*, IL-6 and colony-forming units (CFU). These values are compared with those predicted by model simulation. These data are used to test if the model can reproduce the reported levels of the different variables and, most importantly, their timing (figure 2). None of the papers selected for validation were used to calibrate the model.

Figure 2 exhibits the comparison of simulation results with literature data. One can observe that the simulator correctly captures the timing of cell counts and cytokine levels. Furthermore, CFU dynamics (figure 2*b*) is also accurately captured by the model. Concerning the exact levels of these cells and cytokines, the mismatches between our simulator and the data in figure 2 are within the range of variation between the different experiments reported in the literature (table 1).

**Table 1.** Extended literature measurements of *Aspergillus fumigatus* outcome parameters. All the papers in this table report data in BAL upon 24 h post-infection and inoculated mice with ≈10^7^ conidia. Column 1 shows the reference; column 2 reported measurements of neutrophils; column 3 log_10_ of CFU; column 4 IL-6; and column 5 TNF.

reference	neutrophils	log_10_ (CFU)	IL-6 (pg ml^−1^)	TNF (pg ml^−1^)
Bhatia *et al.* [42]	9.60 ± 0.14 × 10^5^			
Brieland *et al.* [43]		5.56 ± 0.10		3027 ± 194
Cenci *et al.* [45]			348 ± 52	1602 ± 297
Dubourdeau *et al.* [46]			64 ± 18	923 ± 174
Doung *et al.* [44]		6.24 ± 0.16	460 ± 8	
Gresnigt *et al.* [47]	5.42 ± 1.64 × 10^5^		364 ± 47	
Hohl *et al.* [48]	2.30 ± 0.92 × 10^6^			
Teschner *et al.* [49]	4.04 ± 1.25 × 10^5^	4.38 ± 0.38	1964 ± 313	592 ± 48
average ± s.d.	1.05 ± 0.87 × 10^6^	5.39 ± 0.94	676 ± 748	1536 ± 1079

**Figure 3.**
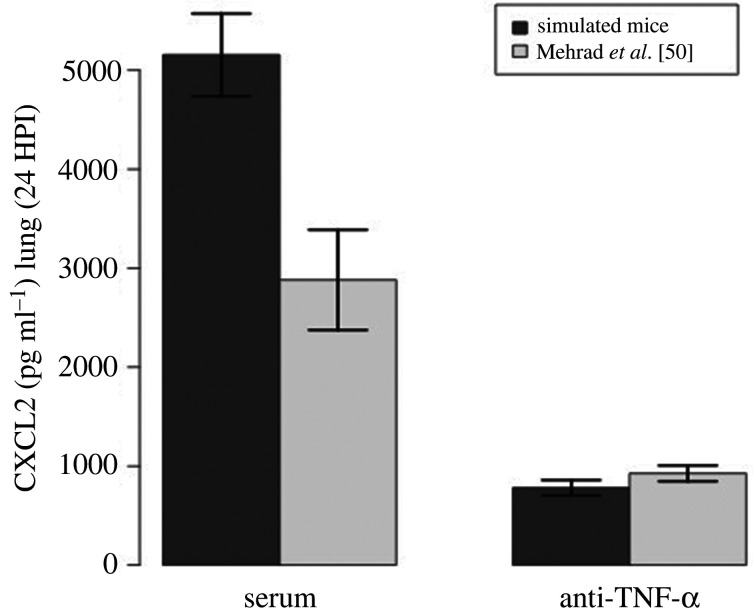
Comparison of simulated data with data reported by Mehrad *et al.* [50]. Mice were injected with unimmunized serum (control) or antibody (anti-TNF-*α*) 24 h before infection. The figure shows lung levels of CXCL2 24 HPI (hours post infection) in mice injected with unimmunized serum (left bars) and with anti-TNF-*α* (right bars). To produce this figure, 36 simulations were performed, initialized with an average of 1920 conidia, 15 macrophages and 640 epithelial cells. To produce the simulation results, we used a concentration of $2\times 10^{-8}\;M$, a reaction rate of 1.72 × 10^8^ M s^−1^ (Kcat/Km), and half-life of 5 days.

Next, we compared model simulations with a published experiment of mice injected with anti-TNF-*α*. This cytokine is one of the critical drivers of the immune response and one of the key molecules inducing the secretion of chemokines. In Mehrad *et al.* [50], the level of chemokines fell 24 h post-infection upon injection with an anti-TNF antibody. To reproduce this experiment, we estimated the affinity of an antibody for a protein antigen with data from the literature [51]. Note that those estimates are for a generic protein antigen and not for TNF specifically. Figure 3 shows that our model correctly predicts the fall in CXCL2 upon injection of anti-TNF-*α*.

We performed an extensive literature search for data concerning *Aspergillus fumigatus* infection in mice. We compiled these data in table 1, representing BAL measurements of IL-6, TNF, CFU and neutrophils 24 h post-infection with 10^7^ conidia. One can observe that these data have significant variability. Table 1 does not take into account the volume of lavage, which could be a possible source of variability in the literature data. However, when we divided the IL-6 and TNF-*α* concentrations by the lavage volume reported by Brieland *et al.* [43], Cenci *et al.* [45], Gresnigt *et al.* [47] and Teschner *et al.* [49] (Dubourdeau *et al.* [46] did not report the volume of lavage and Doung *et al.* [44] reports only that for cells), we found that neither the average nor the variance changed significantly.

**Figure 4.**
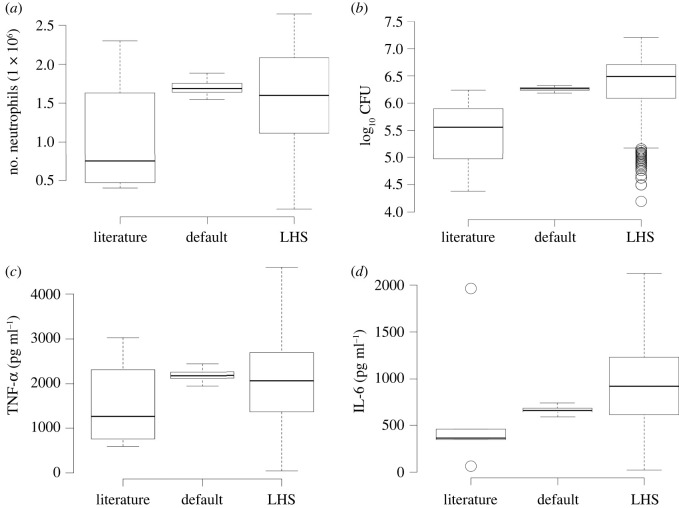
Comparison of simulated data with extended literature reported data (table 1). Literature: data from the literature (table 1). Default: simulator run with default values. LHS: simulator run with parameters sampled with latin hypercube sampling. The ‘default’, is the average of 36 simulations with the default parameter values; see electronic supplementary material, table S1. To generate the ‘LHS’, we ran 1200 simulations with LHS sampled around the default values.

To understand how our simulated data compare to these measurements, we sampled parameters with latin hypercube sampling (LHS) and ran 1200 simulations. Figure 4 displays the comparison between several literature measurements, the simulator with the default parameters (electronic supplementary material, table S1), and the simulator with LHS parameters. The predictions made by our simulator are within the range of variation between data reported in the literature. Note that the simulator can also reproduce the variability in biological data. This variability is due to differences in hosts, different fungal strains and experimental conditions. These conditions sometimes generate different but qualitatively equivalent outcomes. Our simulator is robust to a wide range of parameters, with variability similar to that observed in the literature (figure 4 and table 1).

One point to observe in figure 4 is that the simulations with default values have a narrow scatter with an average near one quartile of the literature data. That is expected since this model was not fitted to data, but was parameterized *a priori* with information from the literature (see electronic supplementary material, table S1) and then compared with novel literature data, that is, papers we did not use to extract parameters. The biggest discrepancy in figure 4 is with CFU (figure 4*b*); however, as we can see in figure 2*b*, when we look at the whole time series, we see that there is strong agreement between simulated CFU and CFU reported in the literature.

#### 3.1.1. *In vivo* validation of simulation results

**Figure 5.**
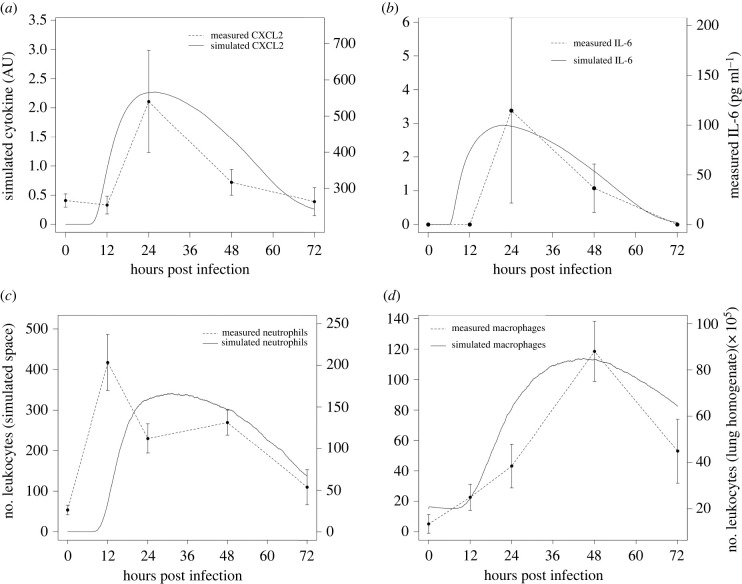
Comparison of our experimental data on immunocompetent mice with simulation results. For this purpose, 36 simulations were performed, starting with an average of 1920 conidia, 15 macrophages and 640 epithelial cells. (*a*) Comparison of simulated time series of CXCL2 with experimental data measured in BAL. (*b*) Comparison of simulated time series of IL-6 with experimental data measured in BAL. (*c*) Comparison of the number of neutrophils in the simulated space with the number of neutrophils in lung homogenate. (*d*) Comparison of the number of macrophages/monocytes in simulated space with the number of monocytes in lung homogenate. Experimental data refer to mice infected with 7 × 10^6^ conidia.

As discussed above, due to the high variability in experimental design (fungal dose, techniques of infection, etc.) and in the measurements summarized in table 1, we decided to further validate the model results, in particular the ability of the simulator to reproduce temporal dynamics, with an *in vivo* experimental design that most closely resembles the simulator setup. We infected immunocompetent mice with 7 × 10^6^ conidia intratracheally and measured cytokines and leucocytes from 0 to 72 h post-infection (see description in §2). Figure 5 shows that our simulator can correctly predict the timing of the immune response, as indicated by levels of IL-6, neutrophils, macrophages and CXCL2.

Figure 5 shows remarkable agreement between the timing of cytokines and cells measured by our own experiments and the dynamics produced by the simulator. To recall, agent-based models are not continuous-time models, since they advance in discrete time steps. The actual time these steps correspond to is estimated by considering the events that take place from one time step to the next. In our case, we estimated that our time steps correspond to 2 min of simulated time. Thus, the agreement in timing is a key step in validating model predictions, since the timing of events in the immune response to this infection is crucial for infection outcome and determination of interventions.

One point to observe in figure 5 is that the neutrophil curve follows the CXCL2 curve. CXCL2 is the molecule responsible for neutrophil recruitment in the simulator and one of the key chemoattractants for neutrophils *in vivo* [50,52]. The spike in figure 5*c* (12 h), is a discrepancy found in this experiment and disagrees with previous experiments in the literature (see [42,44] and figure 2).

### 3.2. Identification of drivers of fungal burden

**Figure 6.**
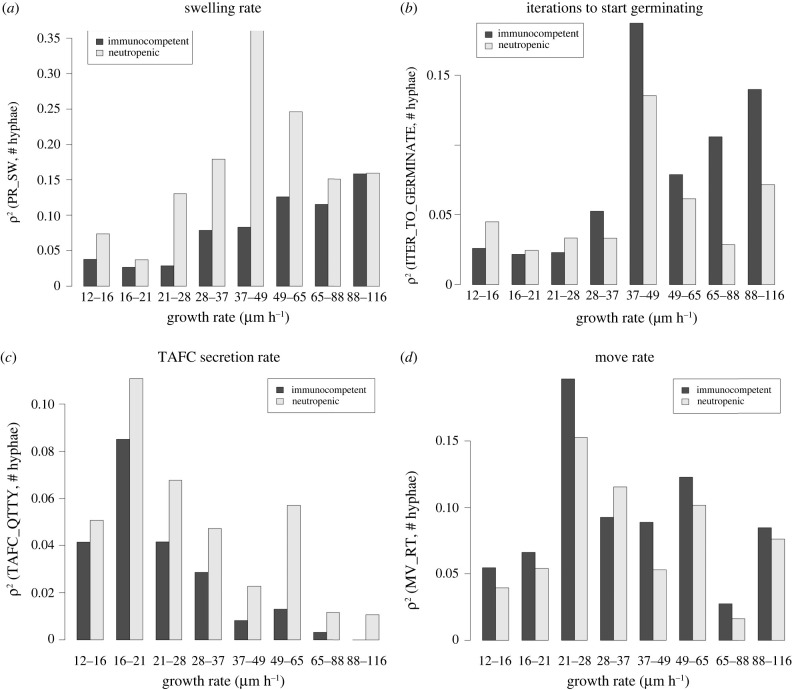
Variation of the square of the correlation *r*^2^ between the critical model parameters and fungal burden, with the variation of the growth rate. We started with a dataset of 1200 samples generated with LHS and then partitioned this dataset by growth rate. The *x*-axis shows the range of the growth rate in each partition. We then calculate the correlation between parameters and fungal burden in each partition. Fungal burden was measured as the maximum number of hyphal cells measured over the course of 24 h of simulation. (*a*) Variation of *r*^2^ between the swelling rate and fungal burden. (*b*) Variation of *r*^2^ between germination rate and fungal burden. (*c*) Variation of *r*^2^ between the TAFC secretion rate and fungal burden. (*d*) Variation of *r*^2^ between the leucocyte movement rate and fungal burden.

We carried out a classification of parameters and their influence on fungal growth rate, encoded by a collection of classification trees in figures S2 and S3 in the electronic supplementary material. The results are summarized in table 2. Our analysis shows that eight model parameters are most strongly correlated to fungal burden. As would be expected, the most critical parameter is the intrinsic growth propensity of a given fungal strain to grow (GR_RT) that remains fixed for a given fungal strain [53]. Fixing this parameter, we then asked which other parameters were associated with high, respectively low, fungal burden over a 24 h period. To do this, we measured the variation of the square of the correlation *r*^2^ between the model parameters and fungal burden as the growth rate increases. Figure 6 and electronic supplementary material, S4, show the variation of *r*^2^ for the seven most important parameters (not including intrinsic growth rate) indicated by our analysis in table 2.

**Table 2.** Table summarizing the classification results from figures S2 and S3. The table shows qualitatively the groups of parameters that lead to the extremes of high and low germination and hyphal proliferation over 24 h of simulated time. Intermediate conditions are not presented here. The plus and minus signs represent, qualitatively, the classifications. For example, in column 4 (immunocompetent, high burden), the three plus signs and one minus sign indicate that the corresponding partition tree (figure S2 in electronic supplementary material) partitioned the dataset by the fungal growth rate, activation rate, swelling rate, and recruitment rate, and selected the upper part of the first three partitions (plus sign) and the lower part of the last (minus sign). In a qualitative sense, this means that class (immunocompetent, high burden) is associated with high growth rate, activation rate, and swelling rate, and with low recruitment rate. The partitioning hierarchy is not represented in this table, as it is meant to be simply a general summary. Some of the parameters shown in the table do not contribute to the extreme cases reported here; they are kept for completeness. These parameters, however, play a role in differentiating intermediate cases of fungal burden (electronic supplementary material, figures S2 and S3).

parameters	parameter description	immunocompetent		neutropenic	
		low burden	high burden	low burden	high burden
GR_RT	growth rate	−	+	−	+
ITER_CH_ST	iterations needed to host cells chance state		+		+
MV_RT	leucocytes movement rate				
REC_RT	leucocytes recruitment rate		−		−
PR_SW	probability of conidia swelling		+		+
TAFC_QTTY	TAFC secretion rate				+
ITER_GER	iterations swelling conidia takes to starts germinating				
PR_BR	probability of hyphae branching				
max. no. hyphae		*28*	*1609*	*133*	*5203*
max. no. hyphae/initial inoculum		*1.5%*	*83.79%*	*6.9%*	*271%*

Figure 6 and electronic supplementary material, S4, show that, as the intrinsic growth rate of the fungus increases, the relative importance of the seven other parameters changes. As an example, figure 6*d* shows that when the fungal growth rate is very high or very low, the leucocyte movement rate is weakly correlated with fungal burden. However, this parameter is critical in determining the actual fungal burden in strains with an intrinsic growth rate near the default 29 μm h^−1^ (electronic supplementary material, table S1). The leucocyte movement rate is an intrinsic feature of the immune system. However, according to figure 6*d*, this feature is more or less critical, depending on the virulence of the strain, being most important for a moderately virulent strain (21–28 μm h^−1^). This might explain some of the variance in the course of the infection between hosts.

Figure 6 shows that overall the seven parameters tend to be more critical when the intrinsic growth rate is close to its default value. That is, their importance wanes as the growth rate becomes very high or very low. However, the importance of the swelling rate, measured as the average number of time steps needed for conidia to germinate, and the average time to immune cell activation have a bias towards a high growth rate (figure 6*a*,*b*,*d*). On the other hand, the TAFC secretion rate has a bias towards a low growth rate (figure 6*c*). Finally, the monocyte recruitment rate is not sensitive to intrinsic growth rate variation (electronic supplementary material, figure S4C).

The results in figure 6 also show that activation, germination and recruitment rates have a bigger influence in immunocompetent hosts (figure 6*b*,*d*; electronic supplementary material, figure S4C). Meanwhile, swelling rate, TAFC secretion rate and branching probability are more crucial in neutropenic hosts (figure 6*a*,*c*; electronic supplementary material, figure S4A). Finally, the leucocyte movement rate is approximately equally important in neutropenic and immunocompetent hosts (figure 6*d*).

## 4. Discussion

Understanding the innate immune response to pathogens is of the utmost importance for designing effective therapeutic interventions. With the increasing resistance of pathogens to anti-microbial drugs, it is imperative to explore host-centric therapeutics. This is the motivation for the work presented here. An essential application of this model is to understand critical features of respiratory fungal infections. The model predicts that certain fungal characteristics, such as the siderophore secretion rate, and immune system characteristics take on different significance in controlling fungal burden, depending on the virulence of the fungal strain in question.

It was pointed out in Schrettl *et al.* [54] that TAFC is a critical virulence factor. Likewise, in subsequent work, Schrettl *et al.* [55] show that an *Aspergillus* knockout strain without intracellular and extracellular siderophores has completely attenuated virulence. Figure 6*c* shows that the TAFC secretion rate is more important when the growth rate is low. In this case, fewer cells are secreting this siderophore. Consequently, the TAFC concentration becomes a bottleneck. In fact, our analysis shows that the concentration of TAFC bound to iron in neutropenic hosts with a high growth rate is 60% higher than in simulations with a low growth rate (simulated result not shown). For immunocompetent hosts, a smaller and less significant difference was observed. However, it is important to observe that both computational and experimental approaches are amenable to false negatives. If there is heme available at the site of inflammation, the fungus might have an alternative source of iron, and there might be little need for the siderophore secretion system.

Interestingly, the classification trees (electronic supplementary material, figure S3; and table 2) show that a high TAFC secretion rate is also necessary for achieving a high fungal burden. That seems to be in accordance with the spike in figure 6*c* (38–49 μm h^−1^). That may indicate a double role for TAFC, where it is crucial when the growth rate is high or low but not intermediate. Note, however, that in electronic supplementary material, figure S3, TAFC acts in synergy with other parameters.

Conidial swelling is the first step to germination and subsequent hyphal growth. However, this is also the time when the fungus become visible to the immune system. Therefore, there are two competing forces upon swelling. The fact that swelling is a necessary step before growth is advantageous for the fungus. This is evidenced by the positive correlation between the swelling rate and fungal burden (results not shown). However, the fact that swelling makes the conidia visible to the immune system and that conidia are easier to kill than hyphae creates a disadvantage for the fungus. This is evidenced by the bias towards a high growth rate (figure 6*a*) in this case. That means that swelling is more advantageous if, upon swelling, hyphae develop quickly. A recent model published by Ewald *et al.* [56] came to similar conclusions.

However, note that in the case of neutropenic simulations, the correlation between swelling and fungal burden peaks at a growth rate of 29−37 μm h^−1^. This indicates an optimal growth/swelling rate relation. The reason is not very clear, but it may be the result of other parameter constraints, such as the TAFC secretion rate. Interestingly, the parameter ITER_GER that controls the time conidia take to germinate after swelling—with 2 h being the default value [55]—has a negative correlation with growth rate (result not shown). This negative correlation reinforces that conidia become susceptible to the immune system and need to germinate rapidly upon swelling.

The ability of leucocytes to locate fungal cells is crucial for controlling the infection [9,11]. The factors that affect it are chemotaxis and movement rate. Figure 6*d* shows that the leucocyte movement rate is one of the most prominent parameters to control fungal burden with *r*^2^ around 0.2 when the growth rate is in the range 21–28 μm h^−1^. However, our analysis also indicates saturation. When the growth rate is too high, the immune system is overwhelmed by fungal growth. Conversely, if the growth rate is too low, the immune system quickly gains the upper hand.

Figure 6 and electronic supplementary material, S4, show the difference between the more relevant parameters for each kind of simulation. One can see that for the immunocompetent simulations, the model is dominated by the immune system parameters: the time host cells take to change status and recruitment rate, as well as the fungal germination rate. In immunocompetent hosts, both in simulations (figure 2*b*; electronic supplementary material, figure S2) and reported in the literature [43,44], the infection tends to be quickly controlled. Therefore, fungal parameters tend to be less critical because the immune system will kill the fungus before it has a chance to grow. Meanwhile, in neutropenic hosts, both simulated (electronic supplementary material, figure S3) and reported [52], the infection progresses and parameters related to the fungus become critical (swelling rate, TAFC secretion rate and branching probability; see figure 6*a*,*c*; electronic supplementary material, figure S4A).

## Data Availability

The model code and all experimental data used for model validation are available in the Github repository https://github.com/NutritionalLungImmunity/IPA_model. The data are provided in electronic supplementary material [57].
